# ‘Are smokers less deserving of expensive treatment? A randomised controlled trial that goes beyond official values’

**DOI:** 10.1186/s12910-015-0019-7

**Published:** 2015-05-04

**Authors:** Joar Björk, Niels Lynøe, Niklas Juth

**Affiliations:** Karolinska Institutet, LIME, Stockholm Centre for Healthcare Ethics, Stockholm, Sweden; Department of Research and Development, Region Kronoberg, Sigfridsvägen 5, Box 1223, 351 12 VÄXJÖ, Kronoberg Sweden

**Keywords:** Health priorities, Physicians, Smoking, Clinical ethics, Decision making

## Abstract

**Background:**

To investigate whether Swedish physicians, contrary to Swedish health care policy, employ considerations of patient responsibility for illness when rationing expensive treatments.

**Methods:**

A random sample of oncologists and pulmonologists made up the main study-group (n = 296). A random sample of GPs (n = 289) and participants from the general population (n = 513) was used as contrast group. The participants randomly received one version of a questionnaire containing a case description of a terminally ill lung cancer patient. The two versions differed in only one aspect: in one version the patient was a smoker and in the other a non-smoker. The main questions were whether to offer a novel, expensive and marginally life-prolonging treatment and whether the patient could be held responsible for her illness. The quantitative data was analysed using Chi2-tests and comments were analysed using content analysis.

**Results:**

Among oncologists and pulmonologists, 78% (95% CI: 72-85) would offer the treatment to the non-smoker and 66% (95% CI: 58-74) to the smoker (Chi-2 = 5.4, df = 1, p = 0.019). Among the GPs, 69% (95% CI: 61-76) would treat the non-smoker and 56% (95% CI: 48-64) the smoker (Chi-1 = 4.9, df = 1 and p = 0.026). Among the general population the corresponding proportions were 84% (95% CI: 79-88) and 69% (95% CI: 63-74).

**Conclusion:**

This study indicates that applying an experimental design allowed us to go beyond the official norms and to show that, compared to a smoking patient, both the general population and physicians are more inclined to treat a non-smoking patient. This clearly runs counter to the official Swedish health care norms. It also seems to run counter to the fact that among the physicians studied, there was no association between finding the patient responsible for her disease and the inclination to treat her. We think these paradoxical findings merit further studies.

**Electronic supplementary material:**

The online version of this article (doi:10.1186/s12910-015-0019-7) contains supplementary material, which is available to authorized users.

## Background

Justice is one of the central virtues in medical ethics, and how to create a just health care system a question of perennial debate [1]. With medical costs rising faster than the capacity to pay, fair priority setting stands out as an increasingly important aspect of health care justice [2].

For a long time, the Rawls-via-Daniels view that health care should be distributed according to patients’ needs but not to deserts or responsibility has been dominant [3]. Most, but not all, health care systems currently operate in accordance with this dictum [4]. In Sweden, a 1996 amendment to the health care law explicitly states that priority-setting based on whether or not a medical condition is self-inflicted – hereafter termed the responsibility principle – is not admissible. The amendment is referred to as the *ethical platform for Swedish priority setting* [5].

This view, however, is not universally accepted. There is now a large body of literature defending the use of the responsibility principle in priority-setting procedures [6-8]. One (but not the only [8]) current defence of the responsibility principle is so-called luck egalitarianism, a view that differentiates between inequalities arising from factors within the individual’s control and those that are beyond the individual’s control. According to luck egalitarianism, justice requires that only the latter and not the former be compensated for.

In some of the literature, a distinction is made between backward-looking and forward-looking principles [9]. A backward-looking responsibility principle claims that patients who have caused their bad health through voluntary but imprudent choices *in the past* should be down-prioritised, required to pay more for medical care, or in some other way stand back to others who have made more prudent choices in the past. A forward-looking responsibility principle, on the other hand, is a consequentialist notion, down-prioritising patients whose *current* imprudent health behaviour might affect *prospective* treatment (e.g. rationing access to liver transplantations for non-abstainers as continued alcohol consumption could damage the new liver). For the purpose of this paper, when we refer to the responsibility principle we mean the backward-looking kind described above.

Medical situations that have been used to illustrate possible applications of the responsibility principle include, apart from liver transplantations in alcohol-related end-stage liver disease [10], various types of cancer due to tobacco smoking [11], obesity due to unhealthy eating habits and physical injury due to extreme sports [8]. Although we know of no medical system fully implementing the idea of personal responsibility in priority setting, it has aroused interest among policymakers in several countries [4]. In Sweden, a 2007 survey report from the National Centre for Priority Setting in Health Care suggested that the responsibility principle be added to the current three principles in the Swedish ethical guidelines for priority setting [12]. However, this proposition met with vigorous opposition [13,14] and the Swedish priority guidelines from 1996 thus far remain unchanged.

Previous empirical research indicates there is some support for the responsibility principle in medical priority setting, especially in the case of tobacco-induced disease [11]. The studies have investigated the views of the general population [15,16] as well as those of physicians [15-17]. Support for the responsibility principle, however, is considerably lower and more contested than support for other priority setting principles, such as the principle of need and the principle of cost-effectiveness [18,19]. When comparing attitudes between the general population and physicians, the latter group seems more reluctant to embrace the responsibility principle than does the general population [15,16,18].

The above-mentioned studies are either interview-based, observational studies using questionnaires or cross-sectional studies where participants sort a list of hypothetical patients in priority order. These methods all bring the prioritisation dilemma to the forefront. Thus, such study designs increase the risk of physicians giving politically correct answers which reflect official policy rather than their personal opinions. To eliminate this risk we have used an experimental design with a prospective, randomised and controlled (blinded) trial which should enable us also to capture such pro-responsibility principle sentiments as physicians might not express when asked directly whether they think responsibility for illness should matter in priority setting. Thus, the aim of this study was to examine whether the method chosen would reveal tacit adherence to responsibility among physicians, primarily by investigating whether or not the studied physicians would be more inclined to give expensive treatment to a non-smoking lung cancer patient than to a smoking patient with the same disease. Members of the general population were used as reference group.

## Methods

### Participants

The group of physicians studied comprised a random sample of 300 oncologists and 300 pulmonologists. From these two groups totally 296 responded. These medical specialities were chosen on the assumption of their being the ones usually treating the kind of patients presented in the questionnaire. As a contrast group we used a random sample of 600 GPs, of whom 289 responded. The random samples were recruited from all over Sweden and drawn from a commercial database of physicians (Cegedim/Stockholm). To check the accuracy of the database, all physicians were asked to specify their main speciality. Of the physicians answering the questionnaire and stating their speciality, 169 were oncologists, 127 were pulmonologists, and 61 did not belong to any of the three specialities we had actually requested. Accordingly, the latter group of physicians were excluded from further analyses. The random sample of the general population included 962 participants, of whom 513 responded. These participants were recruited from the Stockholm area and provided by the tax authorities (SPAR).

The introductory letter stated that participation was optional and we assumed that completion of the questionnaire constituted consent to the study. When calculating the response rates, all questionnaires returned to sender because undeliverable (7 among physicians, 23 among the general population) were left out. For background variables and response rates, see Table 1.

**Table 1:** **Shows background variables relevant to the randomisation of the two versions of the questionnaire**

		**The Smoking patient**	**The Non-smoking patient**
**General population**			
Total number and response-rate		259 (54.8%)	254 (55.6%)
	Sex (M/F)	48.6%/51.4%	47.7%/52.3%
	Age (median (min/max))	50 years (21-82)	52 years (20-80)
*Trust in healthcare was:*	High	84.5%	85.2%
	Low	15.5%	14.8%
*Smoking status:*			
	Current smoker (yes)	7.5%	5.3%
	Stopped smoking (yes)	35.8%	36.9%
	Never smoked (yes)	56.7%	57.8%
**Physicians**			
Numbers and response-rates			
	*GPs*	147 (50.9%)	142 (49.1%)
	*Oncologists*	79 (46.7%)	90 (53.3%)
	*Pulmonologists*	67 (52.8%)	60 (47.2%)
	*All physicians*	293 (50.1%)	292 (49.9%)
Sex (M/F)			
	*All physicians*	49.7%/50.3%	53.7%/46.3%
Median age (median (min/max))			
	*GPs*	59 years (32-74)	59 years (30-76)
	*Oncologists*	53 years (33-78)	51 years (32-75)
	*Pulmonologists*	56 years (35-70)	57 years (35-74)
	*All physicians*	57 years (32-78)	57 years (30-76)
Smoking status (all physicians):			
	Current smoker (yes)	0.3%	1%
	Stopped smoking (yes)	31.8%	32.8%
	Never smoked (yes)	67.9%	66.2%

The study included a random sample of physicians and members of the general population. The questionnaire was randomly provided in two versions differing in only one aspect: in one version the patient was a smoker and in the other a non-smoker.

### The questionnaires

Inspired by Joshua Knobe’s experimental studies on how people ascribe intentions to others’ behaviour, we created two case descriptions that were identical in all aspects but one – the patient’s smoking status – to study how this particular aspect affected responses. Thus, both questionnaires contained a brief case description involving a 59-year-old woman with incurable, disseminated lung cancer. It was stated that there was a novel treatment that would prolong her life approximately 10 weeks. It was further mentioned that this treatment was not routinely offered and that it was expensive, but no specific cost was given. In one version of the questionnaire (see Additional file 1), the patient was stated to be a current smoker with a 40 pack-year history, whereas in the other version the patient had never smoked (see Additional file 2). Both questionnaires concluded with the question whether or not to offer the new treatment (response options: yes/no). We also asked whether or not the patient’s disease was caused by tobacco smoke and whether or not the patient could be held responsible for her disease (response options: yes/no). Finally we asked about age, sex, own smoking habits etc. – see Table 1.

### Randomisation and blinding

The two versions of the questionnaire were randomly distributed to all participants by paper mail, including two reminders. The randomisation was executed within each group, by allotting each participant a list number, giving all even numbers “the smoking patient version” and all odd numbers “the non-smoking patient version”. Thus, within each subgroup (GPs, oncologists, pulmonologists, the general population), the two versions were distributed in equal shares. The introductory letter ignored the existence of another version of the case presentation. In this sense the experiment was blinded to the respondents.

### Statistics

The randomisation procedure resulted in two groups which were similar in all relevant aspects; see Table 1. Thus two independent samples were obtained, and Chi-2 test or Fisher’s exact test were used to calculate p-values. A significance level of 0.05 was chosen. The results were also presented as proportions with a 95% confidence interval (CI), on the assumption that non-overlapping intervals would have resulted in significant differences if a hypothesis-test had been applied. Logistic regression analysis was performed in order to study potential associations and interactions between the main outcome variable and other variables. The data were registered and analysed using the Epi-info software 6.04. When analysing the comments, content analysis [20] was used to register respondents’ stated reasons in support of their choice between offering and not offering the patient the suggested treatment.

### Ethics

The study protocol was approved by the Stockholm Regional research ethics committee (Dn 2014/344-31/2).

## Results

There was a significant difference in response pattern between those who received the smoking patient version and those who received the non-smoking patient version of the questionnaire. As shown in Table 2, both the physicians and the members of the general population were significantly less willing to offer the new and expensive treatment if the patient had been smoking. This difference was larger among the general population (Chi-2 = 15.98; df = 1, p = 0.000064) than among the whole group of physicians (Chi-2 = 10.55; df = 1, p = 0.0012).

**Table 2:** **Difference in inclination to offer treatment across medical specialties**

	**The smoking patient**	**The non-smoking patient**
Oncologists (n = 77) (n = 88)	64.9% (CI 54.2-75.6)	81.8% (CI 73.7-89.9)*
Pulmonologists (n = 67) (n = 59)	67.2% (CI 56-78.4)	72.9% (CI 61.6-84.2)
GPs (n = 144) (n = 136)	56.3 (CI 48.2-64.4)	69.1% (CI 61.3-75.9)*
All physicians (n = 288) (n = 283)	61.1% (CI 55.5-66.7)	73.9% (CI 68.8-79)*
General population (n = 252) (n = 253)	68.7% (CI 63-74.4)	83.8% (CI 79.3-88.3)*

Results are presented as the proportion that would offer the new, expensive treatment among the group that received the smoking patient version and the non-smoking patient version, respectively. Numbers in brackets refer to the amount of respondents having received each version of the questionnaire. 95% confidence intervals (CI) are given. An *means that p < 0.05.

When subgrouping according to medical specialities, we found the above-mentioned disinclination to treat the smoker compared to the non-smoker among oncologists and GPs only (Chi-2 = 6.1; df = 1, p = 0.013 and Chi-2 = 4.9; df = 1, p = 0.026 respectively). Among pulmonologists, there was no significant difference in the inclination to treat depending upon the patient’s smoking status (Chi-2 = 0.49, df = 1 and p = 0.48). See Table 2.

We also analysed the respondents’ own smoking habits and found that, among the general population, current smokers too were significantly less inclined to treat the smoking patient compared to the non-smoking patient (Fisher’s exact two-tailed test, p = 0.02) – the difference was even greater than among non-smoking respondents from the general population; see Table 3. As very few of the physicians stated that they were current smokers (n = 3), this analysis was not feasible in their group.

**Table 3:** **Difference in inclination to offer treatment depending upon respondent’s own smoking status**

	**The smoking patient**	**The non-smoking patient**
Non-smokers (n = 143) (n = 147)	72% (CI 64.8-79.4)	81.6% (CI 75.3-87.9)
Previous smokers (n = 90) (n = 96)	68.9% (59.3-78.5)	86.5% (CI 79.7-93.3)*
Smokers (n = 19) (n = 14)	52.6% (CI 30.1-75.1)	92.9% (CI 79.4-100)*

Results are given for the general population only. Results are presented as the proportion that would offer the new, expensive treatment among the group that received the smoking patient version and the non-smoking patient version, respectively. Numbers in brackets refer to the amount of respondents having received each version of the questionnaire. 95% confidence intervals (CI) are given. An * means that p < 0.05.

Since almost all (95.7%) respondents who received the non-smoking patient version of the questionnaire stated that this patient could not be held responsible for her disease, we focused on the response-pattern of those who had received the smoking patient version for further analysis. In this group, respondents from the general population who found the patient responsible 64.2% (CI: 57.2-71.2) stated that they would offer the new, expensive treatment. Among those who found the smoking patient not responsible for her disease, 82.5% (CI: 72.6-92.4) would offer treatment (Chi-2 = 6.7, df = 1, p = 0.01). In contrast, we found no such differences among the physicians, neither when the whole group was analysed (Chi-2 = 0.00, df = 1, p = 0.97), nor when it was sub-grouped according to medical speciality.

We found no response-pattern associations to participants’ sex and age.

We also analysed the comments on the question of whether to offer the patient the new and expensive treatment. Even though the physicians phrased their answers differently, the manifest content was similar to the answers provided by the general population. Most, but not all, stated reasons pro et contra treatment were also the same, regardless of which version of the questionnaire the respondent had received; see Table 4.

**Table 4:** **Respondents’ stated reasons for and against offering treatment**

	**The smoking patient**	**The non-smoking patient**
Would offer treatment	*Cost does not matter* 21 (16/5)	*Compassion with the patient* 15 (11/4)
	*Ethical considerations* 14 (11/3)	*Cost does not matter* 13 (8/5)
	*Compassion with the patient* 12 (6/6)	*The patient wants it* 10 (8/2)
	*Knowledge gain* 8 (5/3)	*Positive effects of treatment* 7 (3/4)
	*The patient wants it* 7 (3/4)	*Ethical considerations* 4 (3/1)
	*Positive effects of treatment* 3 (2/1)	*Knowledge gain* 4 (2/2)
	*Patient is young* 2 (2/-)	*Patient is young* 1 (1/-)
Would not offer treatment	*Cost matters* 22 (11/11)	*Cost matters* 20 (12/8)
	*Ethical considerations* 3 (3/-)	*Negative effects of treatment* 6 (3/3)
	*Negative effects of treatment* 2 (2/-)	

Comments are grouped by which version of the questionnaire respondents had received. Numbers before brackets refer to total amount of comments; numbers in brackets refer to amount of comments from the general population and physicians, respectively.

## Discussion

The main finding of this study is that in a sample of Swedish physicians as well as the general population, more respondents were willing to offer a new, expensive treatment to a non-smoking lung cancer patient than to a smoking patient with the same disease. Hence, a statistically significantly larger proportion of the studied physicians make a priority decision that seems to be in conflict with the official values expressed in the ethical platform for Swedish priority setting. However, there was no association between perceiving that the smoking patient was responsible for her lung cancer and the inclination to offer her treatment. This is compatible with, and could even be interpreted as indicating support for, the ethical platform. It seems as if the two findings contradict each other: if physicians fully accept the ethical platform and that the principle of responsibility has no place in Swedish priority decision, they ought not discriminate between the smoker and non-smoker in this case.

Our data do not elucidate why this inconsistency of attitudes occurred at group level. One possibility is that a corresponding inconsistency also exists within individual respondents. This thought draws strength from a comparison with studies of peoples’ juridical intuitions, where it has been demonstrated that some people favour more stringent punishment while simultaneously opting for shorter sentence length in case settings [21]. This could be interpreted as a kind of mental compartmentalisation: perhaps people do not realise they are expressing responsibility-based intuition when asked about how to choose in a specific case, even if that is what they are doing. Therefore they can say they accept the official values expressed in laws, platforms, and guidelines, but still make case judgements that are at variance with the same values. Putting it in Rawlsian language: they have failed to achieve a reflective equilibrium in their value system.

The other possibility does not imply failure of reflective equilibrium on an individual level, but rather that the respondents discriminate against the smoker for some reason other than the perception that the she is responsible for her illness. Even so, this reason must be related to the fact that she is a smoker as this is the only difference between the two versions of the questionnaire, and it still contradicts the ethical platform, but not necessarily by supporting the responsibility principle. With either possibility, what remains to be explained is why some physicians harbour such forceful anti-smoker sentiments as to effectively overrule their stated disinclination to let the issue of responsibility for illness govern the decision to treat. More on this later.

In our study, oncologists were more inclined than GPs to offer the new, expensive treatment, regardless of the patient’s smoking status. This might have to do with the difference in perspectives and interests between the two groups of physicians. GPs seldom treat terminal cancer patients and, as a general rule, seldom prescribe very expensive types of medication. Indeed, among the comments from GPs in our material, many stated that tax-payers’ money ought preferably to be used for prevention or in the treatment of curable diseases.

In their comments GPs also expressed that since the patient derives no long-term benefit from the treatment, her needs-based indication for treatment is weak. In the academic discourse, this interpretation of medical need is called the *capacity to benefit* perspective [22]. Although not uncontested, this view is not in apparent conflict with the Swedish priority setting guidelines [5].

Oncologists, on the other hand, have quite a different professional perspective than the GPs, as they are the ones who actually work with this group of patients. Thus, they are closely familiar with the kind of treatment described in the questionnaire. Also, oncologists participate more often than GPs in the development of these kinds of new drugs and drug regimens. Indeed, some comments from oncologists in our material illustrated that they think that when a new treatment exists, it should be used. As one (oncologist) respondent rhetorically stated: why develop new drugs if we aren’t going to use them?

Interestingly, pulmonologists differed from GPs, oncologists and the general population, in that they did not discriminate between the smoking and the non-smoking patient in their inclination to treat. Perhaps this is due to the fact that pulmonologists are very used to working with smoking and ex-smoking patients from their experience with COPD patients. Indeed, several comments from pulmonologists indicated they sought to put the question of responsibility in perspective by discussing the role of the tobacco industry, tobacco addiction and genetic vulnerability.

The above findings are in apparent contrast to the study by Neuberger et al where GPs, gastroenterologists and the general population were asked to rank criteria for patient selection in the case of liver transplantation [15]. In that study only the gastroenterologists – who work just as closely with potential liver transplant patients as oncologists do with lung cancer patients – stated that alcohol consumption should be rated as one of the three most important criteria. However, this difference could very plausibly be explained with reference to consequentialist reasoning. Our study focused on a marginally life-prolonging medication for a dying lung cancer patient, unlike Neuberger’s potentially curative treatment where the gastroenterologists might be concerned with the effect of continued alcohol consumption on the transplant survival.

Regardless of which version of the questionnaire they had received, physicians were less inclined to offer the treatment than were the respondents from the general population. This might indicate physicians have a greater sense of economic awareness regarding medical treatment, or result from the fact that physicians are more used than lay persons to the thought of restricting access to treatment [16].

Interestingly, we found that smoking respondents in the general population were even less inclined to offer treatment to the smoking case patient than were the non-smoking respondents. On first thought, one might expect the smoker subgroup to have been more lenient towards smoking patients, especially since this would be in their own best interest. However, there is a body of literature suggesting a “self-blaming” attitude among smokers that could explain this observation [23].

Why, then, is there such a negative bias against smoking? Obviously, the many health risks, even outside of lung cancer, play a role. Furthermore, comments in the present material indicate that respondents perceive smoking to be a personal choice, of the kind for which the individual should bear the consequences. One further reason for anti-smoking sentiment is based upon notions of (economic) solidarity. This view was also expressed in many comments, but none put is as succinctly as American philosopher Daniel Wikler: “The person who takes risks with his own health gambles with resources which belong to others” [24].

### Implications of the study

If a subgroup of Swedish physicians use smoking status as a criterion in priority setting, as indicated in the present study, this is in violation of the intentions of the Swedish guidelines for priority setting in health care. Interestingly, this violation appears to stem, not from physicians wholeheartedly embracing the responsibility principle – as no association was seen between perception of responsibility for illness and inclination to treat – but possibly from lack of reflective equilibrium or from anti-smoker sentiment as described above. In order to resolve such inconsistencies or anti-smoker sentiment, in-depth discussions among Swedish physicians about the guidelines and their implications are probably needed. Also, observations of clinical practice are needed in order to determine whether these expressed values influence real-world priority setting. Furthermore, we describe a new method of inquiry into case-based decision making that can reveal controversial opinions even in the face of clear official guidelines. It might very well prove fruitful to apply this method to other harmful activities than smoking. Broadening the perspective, we think other questions in applied medical ethics could be addressed using this method.

### Strengths and limitations of the study

The obvious strength of this study lies in the fact that it was conducted within the framework of an experimental, randomised and controlled trial, placing it higher in the evidence hierarchy than previous studies in this field. It was this experimental design that enabled us to reveal the difference between case-specific and official values and norms described above. Furthermore, as the randomisation process resulted in comparable groups regarding relevant aspects, the design minimised the risk of bias.

However, due to the relatively low response-rate among physicians, we cannot know to what degree the results are generalisable. Comments made by the respondents indicate that the physicians found the case description over-simplified, which could be a reason for the low response rate in that group.

The framing of the patient case in the questionnaire, in regard to the cost and expected effect of the new treatment, is also likely to have influenced the overall proportions of both physicians’ and the general populations’ inclination to offer treatment in both cases. Had the cost of the treatment been explicitly stated (and set rather low), more responders would probably have offered treatment – and vice-versa. However, the absolute proportions of those inclined to offer the proposed treatment are not our primary focus. Rather, the core issue is the demonstrated difference across the studied groups regarding inclination to treat the patient depending upon her smoking status.

Among the general population in our sample, there were only 5% current smokers. According to healthcare reports we would have expected rates of around 12-13% [25]. One smoking respondent from the general population stated that, to her, the questions were too sensitive to answer. If that feeling is shared by many smokers, it could help to explain the low inclusion of smokers. Also, there are well known socio-economical associations between smoking and education that render smokers less inclined to answer any questionnaire.

## Conclusions

This study indicates that applying an experimental design allows us to go beyond the official norms and to show that, compared to a smoking patient, both the general population and physicians are more inclined to treat a non-smoking patient. This clearly runs counter to the official Swedish health care norms. It also seems to run counter to the fact that, among the physicians studied, there was no association between finding the patient responsible for her disease and the inclination to treat her. We think these paradoxical findings merit further studies.

## Additional files

Additional file 1:
**The smoking patient version of the questionnaire.**
Additional file 2:
**The non-smoking patient version of the questionnaire.**
