# Supplementary material for: ‘Are smokers less deserving of expensive treatment? A randomised controlled trial that goes beyond official values’
Source: BMC Med Ethics. 2015 May 4;16:28. doi: 10.1186/s12910-015-0019-7 (PMC4425923; doi:10.1186/s12910-015-0019-7)
Supplement: Additional file 1: — The smoking patient version of the questionnaire. [file 12910_2015_19_MOESM1_ESM.doc]

A 59 year old woman has lung cancer since two years. She is a smoker and has been smoking 20 cigarettes a day since the age of 18. This kind of lung cancer is usually associated with cigarette smoking.

After receiving the diagnosis, the patient was put on chemotherapy and radiotherapy. Initially, the treatments seemed effective, but over the last six months the cancer has been spreading and the previous treatment can no longer help her. Instead, she is put on palliative treatment.

However, there is a new, costly treatment option for this kind of lung cancer. Studies show that it can prolong life with up to ten weeks. Without the treatment the patient is expected to live only three more weeks.

The patient is very eager to receive the new treatment, as she wishes to participate at the birthday party of her only grandchild next month.

Although it is possible to offer the treatment at this clinic, it is not yet part of the routine management to do so in a case like this.

Please check the box that best matches your opinion:

1)* Do you think there is a medical indication to use the new treatment in this case?

Yes □ No □

Comments:

2) Do you think the patient should be offered the new treatment?

Yes □ No □

Comments:

3) Do you think tobacco caused this patient’s lung cancer?

Yes □ No □

Comments:

4) Do you think this patient is responsible for her disease?

Yes □ No □

Comments:

5) If a patient such as the one described above would be offered a life prolonging, expensive treatment, it would affect my confidence in health care thus:

My confidence would increase □

My confidence would not change □

My confidence would decrease □

Comments:

6) If a patient such as the one described above would be offered a life prolonging, expensive treatment, I think it would affect the populations’ confidence in health care thus:

The populations’ confidence would increase □

The populations’ confidence would not change □

The populations’ confidence would decrease □

Comments:

**At last, a few questions about yourself:**

Age:_______ I am: Male Female

I smoke: Yes No

I have previously been a smoker Yes No

**Thank you for participating!**

*) NOTE TO THE EDITOR: THIS QUESTION WAS INCLUDED IN THE QUESTIONNAIRE SENT OUT TO PHYSICIANS, BUT NOT IN THE QUESTIONNAIRE SENT OUT TO THE GENERAL POPULATION. THIS WAS THE ONLY DIFFERENCE BETWEEN QUESTIONNAIRES SENT TO PHYSICIANS AND THE GENERAL POPULATION.
